# Access to Essential Medicines for Diabetes Care: Availability, Price, and Affordability in Central Ethiopia

**DOI:** 10.21203/rs.3.rs-3694051/v1

**Published:** 2023-12-04

**Authors:** Hachalu Dugasa Deressa, Habtamu Abuye, Alemayehu Adinew, Mohammed K. Ali, Tedla Kebede, Bruck Messele Habte

**Affiliations:** School of Pharmacy, College of Health Sciences, Addis Ababa University, Addis Ababa, Ethiopia; Department of Pharmacy, College of Medicine and Health Sciences, Wachemo University, Hossaena, Ethiopia; School of Pharmacy, College of Health Sciences, Addis Ababa University, Addis Ababa, Ethiopia; Department of Family and Preventive Medicine, School of Medicine, Emory University, Atlanta, GA; School of Medicine, College of Health Sciences, Addis Ababa University; School of Pharmacy, College of Health Sciences, Addis Ababa University, Addis Ababa, Ethiopia

**Keywords:** access, availability, affordability, diabetes, essential medicines, non-communicable diseases, central Ethiopia

## Abstract

**Background:**

Diabetes is a major global public health burden. Effective diabetes management is highly dependent on the availability of affordable and quality-assured essential medicines (EMs) which is a challenge especially in low-and-middle-income countries such as Ethiopia.

**Methods:**

A cross-sectional study was conducted in 60 selected public and private medicine outlets in central Ethiopia from January to February 2022 using the World Health Organization/Health Action International (WHO/HAI) standard tool to assess access to EMs. We included EMs that lower glucose, blood pressure, and cholesterol as these are all critical for diabetes care. Availability was determined as the percentage of surveyed outlets per sector in which the selected lowest-priced generic (LPG) and originator brand (OB) products were found. The number of days’ wages required by the lowest paid government worker (LPGW) to purchase a one month’s supply of medicines was used to measure affordability while median price was determined to assess patient price and price markup difference between public procurement and retail prices.

**Results:**

Across all facilities, availability of LPG and OB medicines were 34.6% and 2.5% respectively. Only two glucose-lowering (glibenclamide 5mg, metformin 500mg) and two blood pressure-lowering medications (nifedipine 20mg and hydrochlorothiazide 25mg) surpassed the WHO’s target of 80% availability. The median price based on the least measurable unit of LPG diabetes EMs was 1.6 ETB (0.033 USD) in public and 4.65 ETB (0.095 USD) in private outlets, respectively. The cost of one month’s supply of diabetes EMs was equivalent to 0.3 to 3.1 days wages in public and 1.0 to 11.0 days wages in private outlets, respectively, for a typical LPGW. Thus, 58.8% and 84.6% of LPG diabetes EMs included in the price analysis were unaffordable in private and public outlets, respectively.

**Conclusion:**

There are big gaps in availability and affordability of EMs used for diabetes in central Ethiopia. Relevant stakeholders should work to improve access to EMs.

## BACKGROUND

Non-communicable diseases (NCDs) have become a major public health problem worldwide. In 2019, NCDs represented 74.5% out of total deaths that occurred globally of which 47% of NCD mortality in low-and-middle-income countries (LMICs) were premature, i.e., before the age of 70 [1]. Diabetes mellitus (DM), one of the most common NCDs, leads to chronic hyperglycemia which is associated with long-term damage and dysfunction of different organs such as the heart, brain, eyes, nerves, and kidneys. Still, with early diagnosis and treatment, many of the harmful effects of the disease can be delayed or even avoided [2]. Globally, it has become a health challenge due to its high prevalence and its cardiovascular (CV) complications [3]. In 2021, International Diabetes Federation (IDF) estimated that there were 536.6 million adults aged 20–79 years with diabetes. For the same year, IDF estimated 6.7 million deaths due to diabetes or its complications corresponding to 12.2 % of globl mortality from all causes in this age group. The corresponding number of adults with diabetes for Ethiopia is 1.92 million, which places it among the top four countries in the African region [4].

According to the World Health Organization (WHO) package of essentials for non-communicable disease (PEN) interventions in low-resource countries, early diagnosis and the provision of affordable and effective medicines are major strategies in reducing the burden of NCDs [5]. Well-functioning health systems are critical for preventing, controlling, and managing the steadily rising diabetes and for improving health outcomes [6]. Access to affordable essential medicines (EMs) and their availability for patients with DM is very important because such patients need these medications life-long [7]. But access to EMs is multifactorial and associated with rational selection and use of medicines, availability and affordability of medicines, sustainable health care financing and reliable supply system of quality products [8]. Failure in one portion of the framework results in malfunctioning of the other [9]. According to the WHO, nearly 2 billion people have no access to EMs, with many people in Africa facing the problem [10, 11]. Taking initiative, WHO had voluntary set 80% targets for availability of EMs and other health technologies to control major NCDs in health facilities (HFs) by 2025 [12].

Like many LMICs, Ethiopia is facing a devastating burden of increasing NCD morbidity and mortality, especially from DM [13]. As a result, Ethiopia started putting several initiatives such as developing the first national guideline on clinical and programmatic management of major NCDs in 2016 [14]. Studies addressing access to EMs for DM care in the country are however scarce. This study aimed to assess the accessibility of EMs used for diabetes care in central Ethiopia’s public and private medicine outlets with respect to availability and affordability parameters.

## METHODS

### Study area, design, and period

The study was conducted in health facilities operating in central Ethiopia where 15% of the country’s population lives [15]. An institution-based cross-sectional survey was utilized to collect data regarding availability, affordability, and pricing of EMs. Estimates were prepared through collecting and analyzing data using the WHO/Health Action International (WHO/HAI) format from January 1, 2022, to February 30, 2022 [16].

### Study facilities selection

The study area has seven administrative districts. Considering Addis Ababa, the capital of the country, as a center for the study, six districts that can be reached within 1 day, have public health facilities (PHFs) that have provided diabetes care services for at least one year, handle selected EMs for diabetes care, and have pharmacy professionals and physicians to manage the interest of patients with NCDs were selected. According to the standardized WHO/HAI methodology, hospitals and health centers’ outpatient pharmacies from the public sector, and retail pharmacies and drug stores from the private sector (closer to the selected public health facilities) were identified and used as study settings [16].

### Medicine outlets selection

The country’s three-tier system (primary, secondary, and tertiary level categorization of HFs) of healthcare served as a baseline for selecting medicine outlets [17]. Purposively taking one main hospital from the higher level of the framework for each selected study area, the remaining PHFs (2 public hospitals and 2 health centers (HCs)) within three hours of travel from it were randomly selected from the lists of PHFs obtained from the health bureaus of Addis Ababa, Oromia, and Amhara regions for the public sector [16]. Five licensed and private medicine outlets (PMOs) which were proximate to selected PHFs in each study area were also chosen by simple random sampling. In total, 60 medicine outlets were included, 30 from the public and 30 from the private sector.

### Study medicines selection

Thirty-five EMs were identified and selected based on the (i) 2019 WHO 21st list of EMs for adults, (ii) medicines commonly used for treatment of DM and medicines for CV risk management that are listed in the current Ethiopian Essential Medicine List (EML), and (iii) those medicines specified in the WHO core medicine list used for comparative measurement of availability across the countries [11, 18]. Two forms of products were selected and surveyed for each medicine; namely, the originated brand (OB), more specifically the brand-name proprietary product, and the lowest-priced generic (LPG) product, the cheapest generic equivalent that was present at each pharmacy during the time of the survey [16].

### Data collection and analysis

Data collection was adapted from the WHO/HAI’s standardized methodology on measuring medicines availability, prices, and affordability [16]. Three experienced pharmacists were appointed and trained as data collectors for this study. They received one-day training on the study’s purpose, the different names, strengths, and dosage forms of selected medicines, how to complete the data collection form, and how to compute unit costs. Data on the availability of EMs was determined by direct observation: a medicine was considered available if it was on the shelf and ready to be dispensed at the time of the visit. Price data (selling prices of medicines for end users) was recorded for medicines in stock. Public-sector procurement prices were gathered from Ethiopia’s public procurement agency, i.e., Ethiopian Pharmaceutical Supply Services during the previous two years.

For tracking quality of data collection, processing, and statistical analysis, data were entered into a customized MS Excel from the workbook provided as part of the WHO/HAI methodology. All medicine outlets surveyed fulfilled the WHO/HAI recommendation criteria to collect data on the selected 35 medicines (Table 1) [16].

### Definitions of availability, price, and affordability of medicines

The availability of each studied EM was measured by its physical presence in the medicine outlets by their specified strength and dosage form on the survey date. It was determined as the mean percentage (%) availability of individual medicines, availability across groups of medicines, variations between product types such as (LPG vs OBs), and of individual medicines between sectors [19–21]. The current study utilized percentage ranges: 0% - absent—not found in any retail outlet surveyed; <30% - very low—very difficult to find; 30%−40% - low—somewhat difficult to find; 50%−80% fairly high—available in some retail outlet; and > 80% very high—good availability to describe the extent of availability of medicine for diabetes care [19, 20].

Prices for products were taken as unit prices and defined as price per capsule or tablet or vial (least measurable unit). It was computed using the following equation.


$$UnitPrice\;=\frac{\;\mathrm{PriceofPackageofMedicineFound}\;}{\;\mathrm{PackSizeofMedicineFound}\;}$$

Both price lists and prices on the pack of medicine were used to fill in the data for each surveyed medicine physically found in each sampled facility. The prices were converted to US dollars using the buying exchange rate, i.e., 1 USD = 49.1482 Ethiopian Birr (ETB) which was taken from the Ethiopian National Bank website on January 1^st^, 2022, the first day of data collection [22]. In the analysis of price data, both LPG and OB medicines were analyzed separately. The median value of retail price, interquartile price ranges, and minimum and maximum prices were used to describe individual medicine prices in local currency (ETB). Price data of medicines that were found in less than four medicine retail outlets were not included in the price analysis, given the small sample size and low precision of potential estimates.

Affordability was estimated by comparing the total cost required to cover the complete course of standard treatment based on the lowest-paid government worker’s (LPGW) daily wage [16]. Assessment of affordability for standard treatment of each medicine used the defined daily dose (DDD) of each EM, which is the expected average maintenance dose per day for adults and serves as a standard dose unit of measurement [23]. Accordingly, affordability was calculated by applying the following equation.


Treatmentcoursecost=NumberofunitdoserequiredforDDDofEMxMedianUnitpriceofEMxDaysofatreatmentcourse

If the cost of a course of treatment of an anti-diabetic medicine is no more than one day’s wage or income, it is considered affordable. The treatment courses that cost more than one day’s wage were classified as unaffordable. Thus, daily wages were used to express affordability and calculated by dividing the cost of the treatment course by the LPGW’s daily wage. As of January 2022, the Ethiopian Civil Service Authority paid 1409 birr per month or 28.67 USD per month to the LPGW. As a result, the daily wage was calculated by dividing the monthly salary for the previous 30 days, which was ETB 46.97 per day (USD 0.96 per day).

## RESULTS

### Availability of diabetes care EMs

The overall mean availability for LPG diabetes EMs was 34.6% (25.5% in PHFs and 45% in PMOs), and 2.5% (0% in PHFs and 5% in PMOs) for OB diabetes EMs. None of the PHFs stocked OB type medicine while six were found in PMOs. Not all surveyed HFs had all the LPG diabetes EMs. Only 19 and 27 LPG EMs, with percentage availability ranging from 3.3–93.3% and 10–100% in PHFs and PMOs, respectively, were available in at least one or more outlets. In PHFs and PMOs, respectively, the availability of only 4 and 9 LPG medications exceeded the WHO target of 80% availability (Table 1).

**Figure 1:**
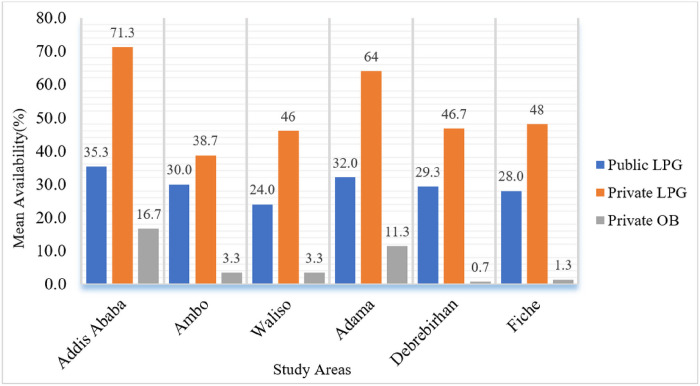
Availability of diabetic care essential medicines by study area

**Figure 2:**
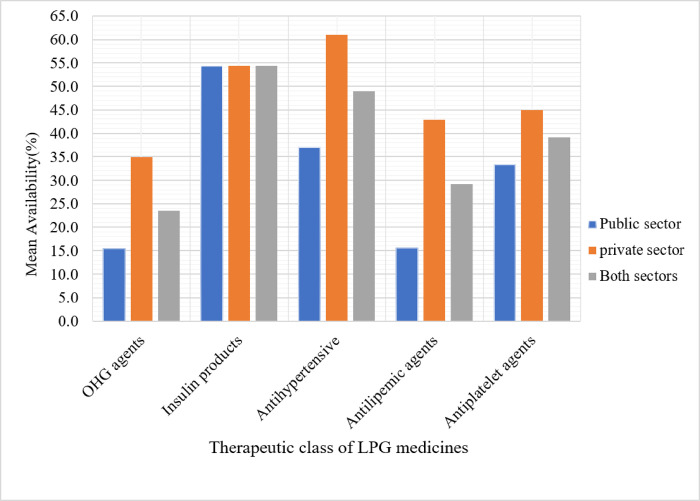
Availability of diabetic care LPG EMs by their therapeutic group

The availability of EMs varied considerably across healthcare levels. From 8 and 17 LPG medications that were found to have ≥ 50% mean availability in PHFs and PMOs respectively, 68.8% accounted for hospitals, 60.6% for pharmacies, 39.4% for drug stores and 31.2% for HCs. Insulin Human Isophane was available in 77.8% of public hospitals but not in any of the HCs. Besides, it was shown that EM availability varied between surveyed areas. Less than 30% of LPG was available in the PHFs of Waliso, Debre Birhan, and Fiche (Fig. 1). PMOs in Addis Ababa and Adama, in contrast, had a stock of EMs with a relatively high percent availability. Furthermore, when the pooled mean availability of LPG was done based on therapeutic classes, as shown in Fig. 2, oral anti-hyperglycemic medications showed relatively poor availability (23.5%). The pooled mean availability of antihypertensive, antilipemic, and antiplatelet medication groups was higher in the PMOs than in the PHFs.

**Figure 3:**
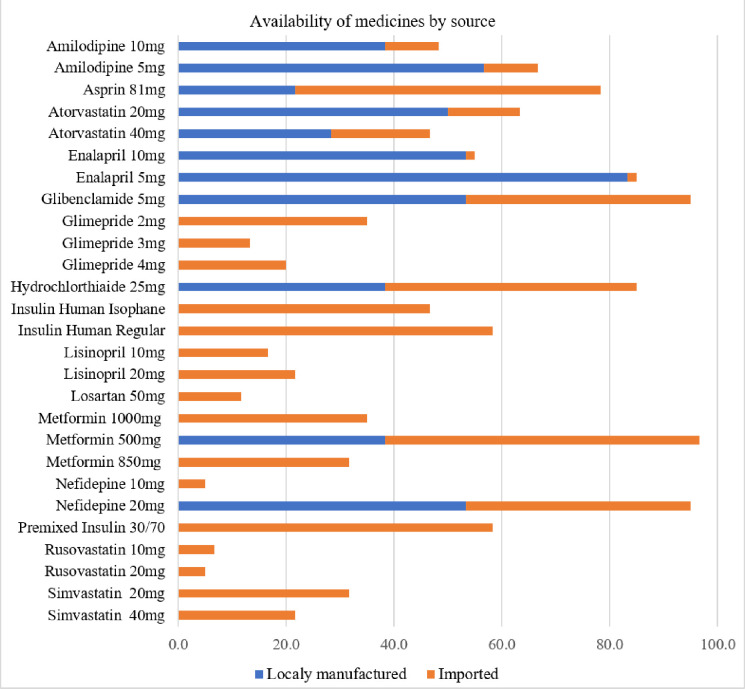
Mean percentage availability of diabetic care LPG EMs medicines by their source (manufacturer)

Availability was impacted by local manufacturing and imports of the surveyed medicines. As shown in Fig. 3, pharmaceutical products that were both manufactured locally and imported from abroad have relatively good availability compared to those that were only imported and not locally manufactured. For instance, the availability of locally produced and imported medicines such as enalapril 5 mg, glibenclamide 5 mg, metformin 500 mg, and nifedipine 20 mg surpassed 85%.

### Price of diabetes care EMs

Seventeen LPG medicines were found in ≥ 4 medicine outlets and hence eligible for measurement of the median price (Table 2). Accordingly, the median prices (based on the medicine price for the least measurable unit) were 1.6 ETB (0.033 USD) and 4.65 ETB (0.095 USD) for PHFs and PMOs respectively. For 16 LPG medicines, patients were paying much higher in PMOs than their public counterparts. For instance, for the second lowest priced medicine among the surveyed EMs— hydrochlorothiazide 25mg tablet, patients paid more than two times as much from PMOs as they would from PHFs (Table 2). Since there were not enough retail outlets where OB-type medicines could be purchased, the median patient price for OB version was not estimated.

In addition, using the public procurement price and the retail price, an inter-sectoral pricing comparison was conducted for 13 LPG medicines commonly found in both sectors. As per the comparison, a markup difference between the patient price and the procurement price registered in the PHFs and PMOs, respectively, was found to be 55.74% and 145.9%. The markup difference between the two sectors was also found to be 56.9%.

### Affordability of diabetes care EMs

The affordability of EMs for diabetes care varied by type of medicine and sector (Table 3). In both sectors, four medicines (glibenclamide 5mg, enalapril 5mg, hydrochlorothiazide 25mg, and amlodipine 5mg) were found to be affordable. All OB medicines observed in PMOs during the study time were found to require payment between 2.9- and 74.1-days’ wages for LPGWs. Eight LPG medicines in PHFs and 18 LPG medicines in PMOs cost more than two days’ wage for a monthly supply of medicines. All Insulin products surveyed across the study area required 3 or more days’ wage for diabetes patients in both sectors. Insulin products were almost 2 times more unaffordable in PMOs than in PHFs.

### Comprehensive analysis of diabetes care EMs availability and affordability

**Figure 4:**
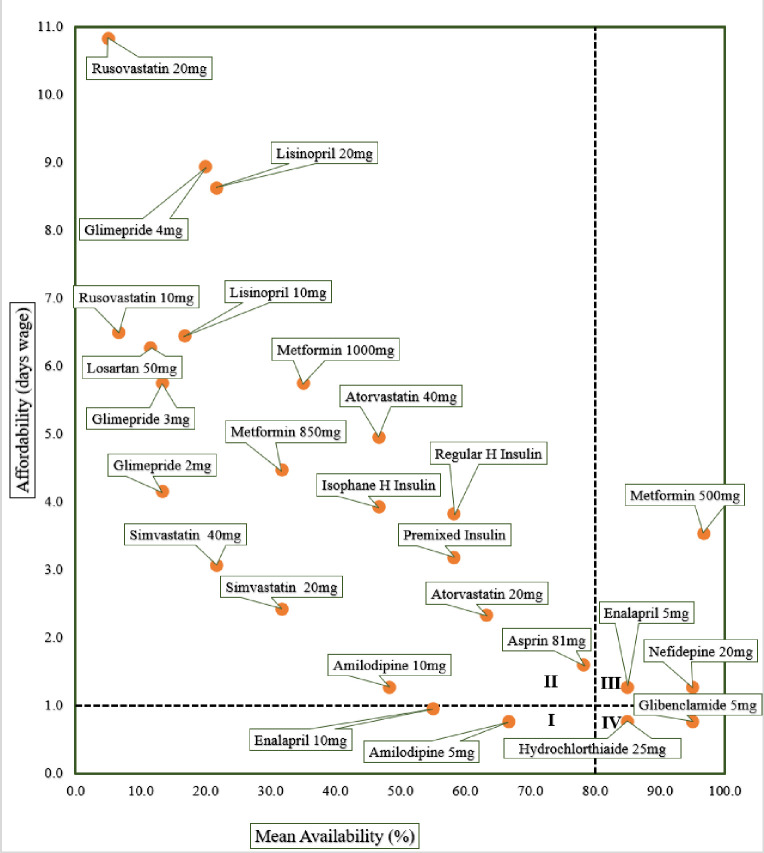
Comprehensive affordability and availability analysis of LPG diabetic care EMs Key: quadrant I: high affordability and low availability., quadrant II: low affordability and very low to fairly high availability., quadrant III: low affordability. and high availability., quadrant IV: high affordability and high availability.

The comprehensive analysis of the availability and affordability of LPG medicines across all surveyed medicine outlets is presented in Fig. 4. The availability score for each medicine is depicted on the X-axis where as the Y-axis was labeled with the number of days wages required to purchase a one-month supply of medicine—affordability. The figure illustrates four quadrants based on WHO/HAI cutpoints: one-day wages for affordability and 80% for high availability. Metformin 500 mg and glibenclamide 5 mg were discovered in Quadrants III and VI, respectively, indicative of good availability although the former required patients to pay more than a day’s wage for a month’s supply. Unfortunately, 73.1% of the medications that were eligible for price analysis were found in Quadrant II, where products’ availability fall behind WHO/HIA availability target and are unaffordable for a typical LPGW.

## DISCUSSION

Access to EMs for the management of DM and CV risk management in terms of availability, patient prices, and affordability was low in all 60 surveyed facilities. The availability of LPGs and OB medicines were 34.6% and 2.5%, respectively, with most medicines well below the WHO-recommended target of 80% availability. The median patient price of LPG diabetes EMs was 0.033 USD in the PHFs and 0.095 USD in the PMOs with most LPG diabetes EMs being unaffordable in both the PHFs and PMOs based on a typical LPGWs’ daily wages.

Our findings are consistent with previous national assessments indicative of low availability of EMs for DM and other NCDs, which is one major determinant for poor glycemic control, and associated morbidity and mortality among patients with diabetes in Ethiopia. While there could be different reasons for the observed low availability, some of the reported ones include lack of attention to NCDs, limited financial resources, and weak supply systems including staff capacity and logistics management information systems, among others [24, 25]. Nevertheless, the finding is a further warning for the healthcare system to address EM availability for DM and related conditions.

Insulin is required for the survival of people with type 1 diabetes and for the enhanced control of diabetes in some patients with type 2 diabetes. It is listed as an EM in the WHO EM list as well as in the Ethiopian EML, demanding that it should be available at all the times [11, 18]. In this study, the availability of insulin products was relatively better compared to oral DM agents and was more available than previous reports from Ethiopia suggest [26–28]. The availability of insulin is comparable to those reported from other LMICs such as Uganda and Brazil [12, 29, 30].

The findings also revealed that the availability of insulin was much better in hospitals (85.2%) and pharmacies (65%) compared to HCs (8.3%) and drug stores (33.3%), similar to findings reported by M. Ewen *et al* [31]. This low availability in or near primary healthcare facilities which serve the majority of the population may lead to unequitable access to EMs for patients with diabetes [32]. This could lead to patients forgoing care at nearby primary healthcare facilities and travelling longer distances to hospitals and pharmacies due to the lack of EMs in nearby outlets. This could create additional barriers to access and in turn lead to lower adherence to follow up and medications [14]. This calls for efforts to ensure distribution of EMs to locations that are accessible to patients with DM.

The median price of 26 LPG medicines was 4.65 ETB in PMOs while that of 17 LPG medicines was 1.6 ETB in PHFs which indicates products in PMOs were sold at three times of median price in PHFs. Price being a key determinant of affordability, it may have indirect role in ensuring access [33]. As the cost of medicines take up the major share for DM and CV risk factors management services, developing policies that address medicine prices for patients and enhance access are critical. Some of these include introducing mechanisms to make pricing transparent, reduce medicines prices such as tax reduction and regulate prices [12].

Most EMs in both sectors were considered unaffordable, costing between 1.2- and 3.2-days’ and 1.3- and 74.1-days’ wage to cover a month’s treatment in the PHFs and PMOs respectively. When EMs remain unaffordable, patients may forgo their treatment especially if they are paying out-of-pocket for their medicines, which increases the burden of DM and its complications [34]. In order to enhance access to EMs and protect citizens from the devastating diabetes complications and financial risk that have huge effect on public health, the country should work to make health care financing sustainable for its efficient operation. These may include expanding the existing community-based health insurance, initiate the social health insurance and introduce other types of health insurance [12, 35, 36].

### Study Limitations

This study has some limitations. Among these is the cross-sectional nature of the study whereby the availability of data reported in this study was based on a one-day visit to surveyed medicine outlets. Hence, it is unable to reflect the average monthly or annual, or overtime availability of medicines at the outlets. Moreover, medicines median price ratios for international price comparison are not reported in this study as the existing international price reference guideline was outdated. Lastly, the affordability of medicines reported was determined by the government’s lowest salary scale for an unskilled worker. Thus, this implies that medications that appear to be relatively affordable in this study may be unaffordable when other expenditures are considered. Therefore, all these limitations need to be considered while generalizing the outcomes of this study.

This study can however provide an important and clear picture to national policymakers on access to EMs for DM and other non-communicable diseases. Different strengths and dosage forms of specific medications were included in this study to circumvent WHO/HAI availability underestimation which occurs by the inclusion of limited strength of medicines. The clinical importance of surveyed medicines has been triangulated between the national EML, the WHO EML, and national and international standard treatment guidelines.

## CONCLUSION

The mean availability of LPG EMs used for diabetes care in central Ethiopia falls far below the WHO target of 80% and the median patient prices for most of the EMs were unaffordable in both PHFs and PMOs based on the LPGWs’ daily wages.

Based on the findings, this study recommends increased government attention to availing affordable EMs for diabetes care. This may require strengthening EMs supply systems, expanding and strengthening financing sources, and pricing mechanisms to mitigate and avoid catastrophic expenditures. Given that these findings are limited to Central Ethiopia, it is recommended that similar studies be conducted in different parts of the country.

## Figures and Tables

**Table 1:** Availability of diabetes care essential medicines (EMs) in the central Ethiopia

EMs name, strengths and dosage form	Percentage of outlets where anti-diabetic and CV risk management EMs were found								
	Public Health Facilities (n=30)			Private Medicine Outlets (n=30)					
	LPG medicines			LPG medicines			OB medicines		
	Hospital (n=18)	HC (n=12)	Total	Pharmacy (n=20)	D/Store (n=10)	Total	Pharmacy (n=20)	D/Store (n=10)	Total
Glibenclamide 5mg tablet	88.9	100	93.3	100	90	96.7	45	10	33.3
Metformin 500mg tablet	88.9	100	93.3	100	100	100	0	0	0
Metformin 850mg tablet	0	0	0	70	50	63.3	0	0	0
Metformin 1000mg tablet	0	0	0	75	60	70	0	0	0
Gliclazide 30mg tablet	0	0	0	0	0	0	0	0	0
Gliclazide 40mg tablet	0	0	0	0	0	0	0	0	0
Gliclazide 80mg tablet	0	0	0	0	0	0	0	0	0
Glimepiride 1mg tablet	0	0	0	0	0	0	20	0	13.3
Glimepiride 2mg tablet	22.2	0.0	13.3	75	20	56.7	50	10	36.7
Glimepiride 3mg tablet	0	0	0	40	0	26.7	0	0	0
Glimepiride 4mg tablet	0	0	0	55	10	40	45	0	30.0
Dapagliflozin 10mg tablet	0	0	0	0	0	0	30	10	23.3
Glucagon 1mg/1ml injection	0	0	0	0	0	0	0	0	0
Insulin Short Acting injection	88.9	8.3	56.7	70	40	60	0	0	0
Insulin medium Acting injection	88.9	16.7	60.0	70	30	56.7	0	0	0
Insulin long acting injection	77.8	0.0	46.7	55	30	46.7	0	0	0
Vildagliptin 50mg tablet	0	0	0	0	0	0	0	0	0
Simvastatin 20mg tablet	16.7	8.3	13.3	65	20	50	0	0	0
Simvastatin 40mg tablet	5.6	8.3	6.7	50	10	36.7	0	0	0
Atorvastatin 20mg tablet	50	33.3	43.3	90	70	83.3	0	0	0
Atorvastatin 40mg tablet	44.4	8.3	30	70	50	63.3	0	0	0
Rosuvastatin 10mg tablet	0	0	0	20	0	13.3	0	0	0
Rosuvastatin 20mg tablet	0	0	0	15	0	10	0	0	0
Losartan 50mg tablet	0	0	0	35	0	23.3	0	0	0
Enalapril 5mg tablet	72.2	83.3	76.7	90	100	93.3	0	0	0
Enalapril 10mg tablet	33.3	16.7	26.7	80	90	83.3	0	0	0
Hydrochlorothiazide 25mg tablet	88.9	91.7	90	85	70	80	0	0	0
Lisinopril 10mg tablet	5.6	0	3.3	45	0	30	0	0	0
Lisinopril 20mg tablet	22.2	8.3	16.7	40	0	26.7	0	0	0
Acetylsalicylic acid 81mg tablet	88.9	33.3	66.7	95	80	90	0	0	0
Acetylsalicylic acid 100mg tablet	0	0	0	0	0	0	45	20	36.7
Nifedipine 10mg tablet	0	0	0	15	0	10	0	0	0
Nifedipine 20mg tablet	88.9	100	93.3	95	100	96.7	0	0	0
Amlodipine 5mg tablet	50	33.3	43.3	95	80	90	0	0	0
Amlodipine 10mg tablet	22.2	16.7	20	80	70	76.7	0	0	0
Overall mean availability (%)	29.8	19	25.5	50.7	33.4	45	6.7	1.4	5

**Table 2:** Median Price [the 25^th^ -75^th^ Percentile] of LPG diabetes care EMs (PHFs, n = 17; PMOs, n=26) *

List of EMs available in at least four medicine outlets	PHFs	PMOs
Glibenclamide 5mg tablet	0.49 [0.30–0.75]	0.60 [0.30–3.00]
Metformin 500mg tablet	0.72 [0.58–2.20]	1.39 [0.58–3.80]
Metformin 850mg tablet		3.50 [1.40–5.33]
Metformin 1000mg tablet		4.50 [3.50–12.50]
Glimepiride 2mg tablet	4.25 [4.00–5.33]	6.50 [4.00–12.00]
Glimepiride 3mg tablet		9.00 [4.00–13.00]
Glimepiride 4mg tablet		14.0 [9.20–15.00]
Insulin Short Acting injection	144.50 [125–150]	180 [125–380]
Insulin medium Acting injection	147.25 [133–150]	150 [133–400]
Insulin long-acting injection	141.70[127.4–184.51]	184.51 [120–400]
Simvastatin 20mg tablet	3.49 [3.00–4.00]	3.80 [3.00–9.50]
Simvastatin 40mg tablet		4.80 [4.10–24.50]
Atorvastatin 20mg tablet	3.40 [2.00–4.50]	3.65 [2.00–23.35]
Atorvastatin 40mg tablet	4.95 [4.80–5.80]	7.75 [4.00–26.50]
Rosuvastatin 10mg tablet		10.18 [10.0–12.0]
Rosuvastatin 20mg tablet		16.95 [15.50–18.50]
Losartan 50mg tablet		9.80 [8.36–11.80]
Enalapril 5mg tablet	0.93 [0.60–2.55]	1.00 [0.60–4.30]
Enalapril 10mg tablet	1.33 [1.00–1.70]	1.50 [1.00–7.50]
Hydrochlorothiazide 25mg tablet	0.50 [0.35–1.30]	1.20 [0.35–2.5]
Lisinopril 10mg tablet		10.10 [1.80–13.00]
Lisinopril 20mg tablet	4.25 [2.10–110]	13.50 [2.10–17.00]
ASA 81mg tablet	0.80 [0.45–3.05]	2.50 [0.45–3.50]
Nifedipine 20mg tablet	0.78 [0.60–1.70]	1.00 [0.50–9.80]
Amlodipine 5mg tablet	0.95 [0.50–1.50]	1.20 [0.50–7.80]
Amlodipine 10mg tablet	1.60 [1.00–2.30]	2.00 [1.00–12.20]
Overall median price	1.60 [0.3–184.51]	4.65 [0.30–400.0]

* — Median Price Ratio (MPR) for international price comparison were not reported in this study as MSH 2015 international price reference (IPR) guideline was outdated to use. Therefore, the study took Health Action International’s recommendation to present the price outcome by median price in the local currency, ETB (0.0203 USD).

**Table 3:** Affordability of EMs for diabetic care: money needed to cover a monthly treatment against days’ wage of LPGW

EMs name, strength and dosage form	DDD	Total*	Product type	Public sector		Private Sector		Over all	
				MTP	Days’ wage	MTP	days wage	MTP	Days’ wage
Glibenclamide 5mg tab	10mg	60	LPG	29.55	0.6	48	1.0	36	0.8
			OB			630	13.4	630	13.4
Metformin 500mg tablet	2g	120	LPG	86.4	1.8	180	3.8	166.2	3.5
Metformin 850mg tablet	2g	60	LPG			210	4.5	210	4.5
Metformin 1000mg tablet	2g	60	LPG			270	5.7	270	5.7
Glimepiride 1mg tablet	2mg	60	OB			780	16.6	780	16.6
Glimepiride 2mg tablet	2mg	30	LPG	127.5	2.7	210	4.5	195	4.2
			OB			699.90	14.9	699.90	14.9
Glimepiride 3mg tablet	2mg	30	LPG			294	6.3	270	5.7
Glimepiride 4mg tablet	2mg	30	LPG			450	9.6	420	8.9
			OB			1125	24	1125	24
Dapagliflozin 10mg tablet		30	OB			3479.9	74.1	3479.9	74.1
Insulin Short Acting injection	40IU	1	LPG	144.5	3.0	290	6.2	180	3.8
Insulin medium Acting	40IU	1	LPG	147.25	3.1	320	6.8	150	3.2
Insulin long Acting	40IU	1	LPG	141.7	3.0	300	6.4	184.5	3.9
Simvastatin 20mg tablet	20mg	30	LPG	104.7	2.2	114	2.4	114	2.4
Simvastatin 40mg tablet	20mg	30	LPG			240	5.1	144	3.1
Atorvastatin 20mg tablet	30mg	30	LPG	102	2.1	120	2.6	109.5	2.3
Atorvastatin 40mg tablet	30mg	30	LPG	148.5	3.1	240	5.1	232.5	4.9
Rosuvastatin 10mg tablet	10mg	30	LPG			305.49	6.5	305.5	6.5
Rosuvastatin 20mg tablet	10mg	30	LPG			516	11.0	508.5	10.8
Losartan 50mg tablet	50mg	30	LPG			294	6.3	294	6.3
Enalapril 5mg tablet	10mg	60	LPG	55.8	1.2	72	1.5	60	1.3
Enalapril 10mg tablet	10mg	30	LPG	39.75	0.8	45	1.0	45	1.0
HCT 25mg tablet	25mg	30	LPG	15	0.3	45	1.0	36	0.8
Lisinopril 10mg tablet	10mg	30	LPG			330	7.0	303	6.5
Lisinopril 20mg tablet	10mg	30	LPG	127.5	2.7	412.5	8.8	405	8.6
Acetylsalicylic acid 81mg tablet	81mg	30	LPG	24	0.5	75	1.6	75	1.6
Acetylsalicylic acid 100mg tablet	100mg	30	OB			135	2.9	135	2.9
Nifedipine 20mg tab	30mg	30	LPG	46.5	1	72	1.5	60	1.3
Amlodipine 5mg tab	5mg	30	LPG	28.5	0.6	45	1.0	36	0.8
Amlodipine 10mg tab	5mg	30	LPG	48	1.0	60	1.3	60	1.3
Mean affordability of LPG					1.75		4.56		4
Mean affordability of OB							24.31		24.31

Total*—Total unit for 30 days treatment; DDD— defined daily dose; MTP—Median treatment price; when data was absent, the cells of table was shaded; LPGW— lowest-paid unskilled government worker’s daily salary; EMs— essential medicines.

## Data Availability

The datasets used and/or analyzed during the current study are available from the first author on reasonable request.

## List Of Abbreviations

CV: Cardiovascular
DDD: Defined Daily Dose
DM: Diabetes mellitus
EMs: Essential Medicines
EML: EM List
ETB: Ethiopian Birr
HCs: Health Centers
IDF: International Diabetes Federation
LMICs: Low-and-Middle-Income Countries
LPG: Lowest-priced Generic
LPGW: Lowest-paid Government Worker
NCDs: Non-communicable diseases
OB: Originated Brand
PHFs: Public Health Facilities
PMOs: Private Medicine Outlets
WHO: World Health Organization
WHO/HAI WHO/: Health Action International
